# Exploration of common genomic signatures of systemic juvenile rheumatoid arthritis and type 1 diabetes

**DOI:** 10.1038/s41598-023-42209-8

**Published:** 2023-09-13

**Authors:** Jie Zheng, Yong Wang, Xin Fang, Jun Hu

**Affiliations:** Department of Pediatric, FuJian Medical University Union Hospital, Fuzhou, 350001 China

**Keywords:** Innate immunity, Functional clustering, Paediatric rheumatic diseases, Type 1 diabetes

## Abstract

To explore the genetic characteristics of systemic juvenile rheumatoid arthritis (sJRA) and type 1 diabetes mellitus (T1D). The microarray data of sJRA and T1D from Gene Expression Omnibus (GEO) were analyzed. The shared differentially expressed genes (SDEGs) were identified by the Meta-analysis, and genes of extracellular proteins were identified. Then, transcription factors (TFs) and their target genes in SDEGs were obtained by comparing databases from HumanTFDB, and hTFtarget. After that, functional enrichment analyses of the previously identified gene sets were performed by metascape tool. Finally, immune infiltration was analysed by CIBERSORT. We found 175 up-regulated and 245 down-regulated SDEGs, and by constructing a TFs-targeted SDEGs network, 3 key TFs (ARID3A, NEF2, RUNX3) were screened. Functional enrichment analyses and immune infiltration results suggested not only the adaptive immune system but also the innate immune system, and signaling pathways like JAK-STAT are important in the pathogenesis of sJRA and T1D, involving biological processes such as CD4 T cell functions and neutrophil degranulation. This work suggests that innate immune abnormalities also play important roles in sJRA and T1D, CD4 T cell functions, neutrophil degranulation and the JAK-STAT pathway may be involved. The regulatory roles of ARID3A, NEF2, and RUNX3 in this network need to be further investigated.

## Introduction

Juvenile idiopathic arthritis (JRA) is the most common chronic rheumatic disease in children, including a group of diseases with different genetic backgrounds, disease courses, and outcomes. Due to its large heterogeneity and unknown etiology, its classification criteria are still constantly improving with the ongoing recognition of the disease^1^. Systemic juvenile idiopathic arthritis (sJRA) is the most serious subtype due to higher disability rates, multisystem involvement, and complications like macrophage activation syndrome, pleuritis, pericarditis, and so on. It is easy to miss diagnosis or misdiagnosis due to its diverse and atypical early clinical manifestations.

Type 1 diabetes (T1D) is one of the major pediatric endocrine diseases, accounting for about 90% of the total diabetes mellitus in childhood. Nowadays, the incidence of T1D tends to be younger, which makes the impact of chronic complications greater (including the risk of death). Moreover, the proportion of acute complications in T1D patients in China is high. For example, the incidence of diabetic ketoacidosis within half a year after diagnosis is as high as 40.1%, which seriously affects the health of Chinese children^2^.

A previous study by Schenck et al. showed a significantly higher prevalence of JRA in T1D patients, however, none of the JRA patients was found complicated with T1D after being treated with anti-rheumatic drugs^3^. On the other hand, Hermann et al. found that the prevalence of JRA in T1D patients was also significantly higher than that in the general population. Furthermore, the patients complicated with JRA had lower hemoglobin A1C levels, which might be owing to the therapeutic control of JRA^4^. Tuller et al. used high-throughput sequencing to explore the gene expression of JRA, T1D, multiple sclerosis, systemic lupus erythematosus(SLE), Crohn's disease, and ulcerative colitis patients and found that T1D and JRA had relatively different gene expression characteristics compared with multiple sclerosis, SLE, Crohn's disease and ulcerative colitis^5^. That evidence suggested that there should be common underlying mechanisms between JRA and T1D. Therefore, exploring the potential shared genetic gene characteristics and possible common pathogenesis of JRA and T1D is not only helping to further understand both diseases but also of important value for the early screening of people at high risk of comorbidity of both diseases.

Peripheral blood mononuclear cell (PBMC) is composed of lymphocytes and monocytes and, thus, play key roles in immune function and autoimmune diseases. The analyses of PBMC help to identify gene expression characteristics associated with immune disorders. Hence, gene sets from PBMC were selected. Given the diversity and heterogeneity among different subtypes of JRA, we focused on exploring the relationship between sJRA and T1D.

## Materials and methods

### Download and process of GEO gene sets

We used the Medical Subject Headings (MeSH) "Arthritis, Juvenile Rheumatoid" or "Diabetes Mellitus Type 1" to search the JRA and T1D gene expression profiles from the GEO database. Gene sets are filtered by: first, the gene expression profile database must include sJRA, T1D, and control groups. Second, the tissue source of origin was the PBMC. Third, these gene sets must contain analytical data, as shown in Table 1.

**Table 1:** GEO datasets involving systemic juvenile rheumatoid arthritis and type 1 diabetes.

GSE number	Sequencing platform	Sample book	Sample type
GSE7753	GPL570	17 sJRA patient, 30 healthy controls	PBMC
GSE21521	GPL570	18 sJRA patient, 29 healthy controls	PBMC
GSE193273	GPL20844	20 T1D patients, compared with 12 healthy controls	PBMC
GSE55100	GPL570	12 T1D patients, 10 healthy controls	PBMC
GSE9006	GPL96	43 T1D patients, 24 healthy controls	PBMC

### Identification of the shared differentially expressed genes by meta-analysis

Meta-analysis of samples in different gene sets was conducted with the R package "ExpressAnalystR"^6^. GSE7753, GSE21521, GSE193273 and GSE55100 were selected for meta-analysis. The individual analysis of each dataset was carried out using Benjamini–Hochberg’s False Discovery Rate (FDR) with cut-off p-values of < 0.05. The microarray chip identifiers were annotated to the official gene symbol, and datasets were merged after annotation. To ensure an unbiased comparative analysis of the different datasets, the batch effect was adjusted through the ComBat batch effect method integrated into the package and was investigated before and after adjustment through principle component analysis. The size effect method was used to identify DEGs between the cases and controls. Fisher's method was used to identify DEGs, according to the instructions. A discovery significant value of < 0.05 was used to identify DEGs.

### The common transcription factors-target SDEGs network construction

A list of 1665 transcription factors (TFs) was downloaded from HumanTFDB to screen out the TFs in the SDEGs (Supplementary Table S1). Potential target genes of TFs were identified in SDEGs by hTFtarget, a database integrating huge human TF target resources including ChIP-seq, motif evidence and epigenetic modification information to predict accurate TF-target regulations^7^. Only those who have all epigenomic, ChIP-seq, and motif evidence were identified as targets (Supplementary Table S2). TFs-target SDEGs network was constructed by Cytoscape (3.9.0).

### Functional enrichment analysis of gene sets

We use metascape (http://metascape.org), a portal based on the latest high-quality data and leverage over 40 independent knowledge bases. It helps to conduct functional enrichment, interactome analysis, gene annotation, and membership search in concerning genes^8^. Accordingly, we performed the enrichment analysis of pathways from the database of Gene Ontology (GO), Kyoto Encyclopedia of Genes and Genomes (KEGG), Reactome (REAC), and WikiPathways (WP), etc.

### Immune cell infiltration analyses

CIBERSORT is a method using the principle of linear support vector regression to deconvolute the expression matrix of 22 human immune cell subtypes with the help of standard LM22 gene signature. To analyze immune cell infiltration in sJRA and T1D samples, the R package "CIBERSORT" was used. We select GSE7753 and GSE9006 to conduct immune infiltration analysis as CIBERSORT requires microarray data that has not been log2 transformed^9^.

## Results

### Characteristic of differentially expressed genes in sJRA and T1D

**Figure 1:**
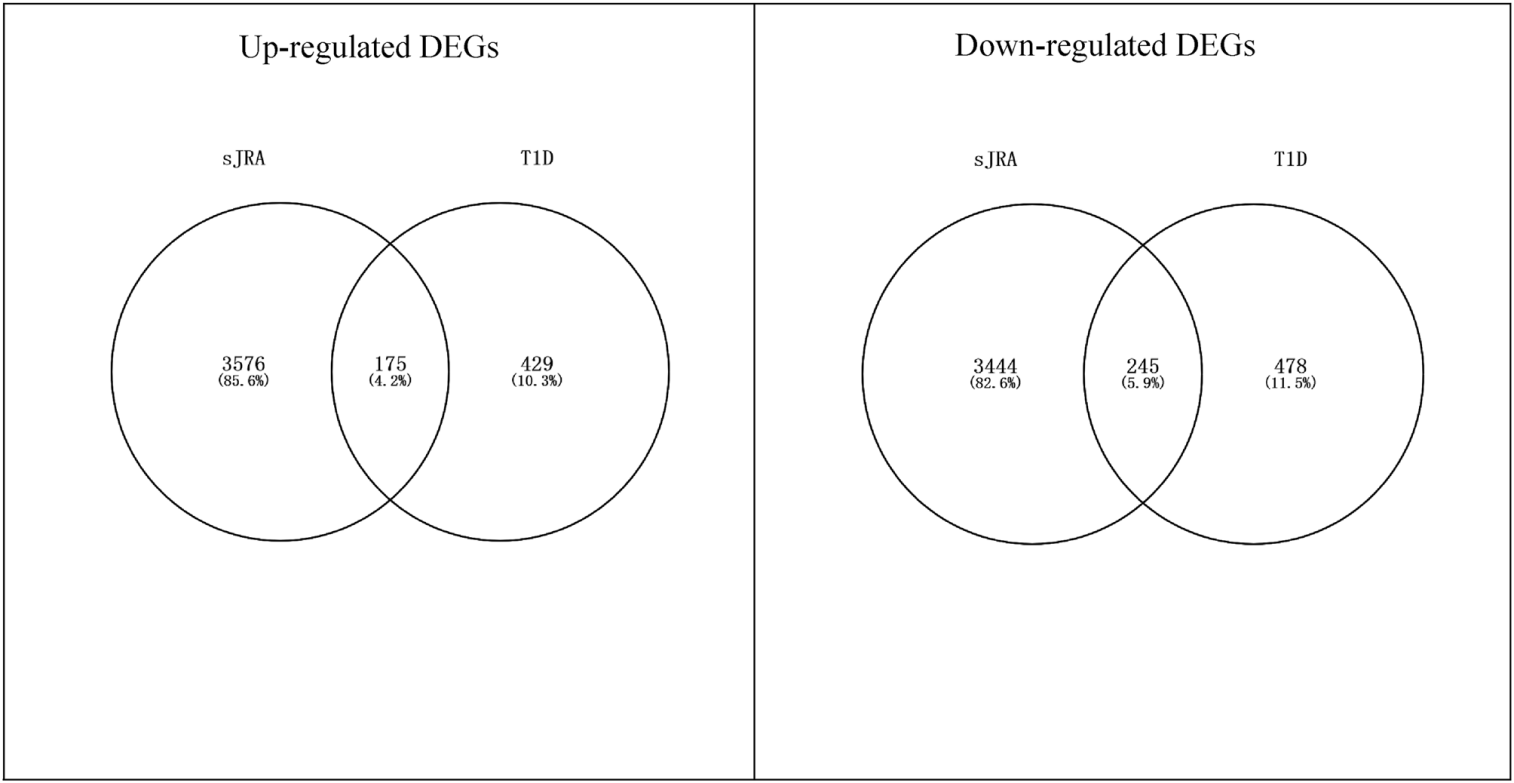
Shared differentially expressed genes in sJRA and T1D.

**Figure 2:**
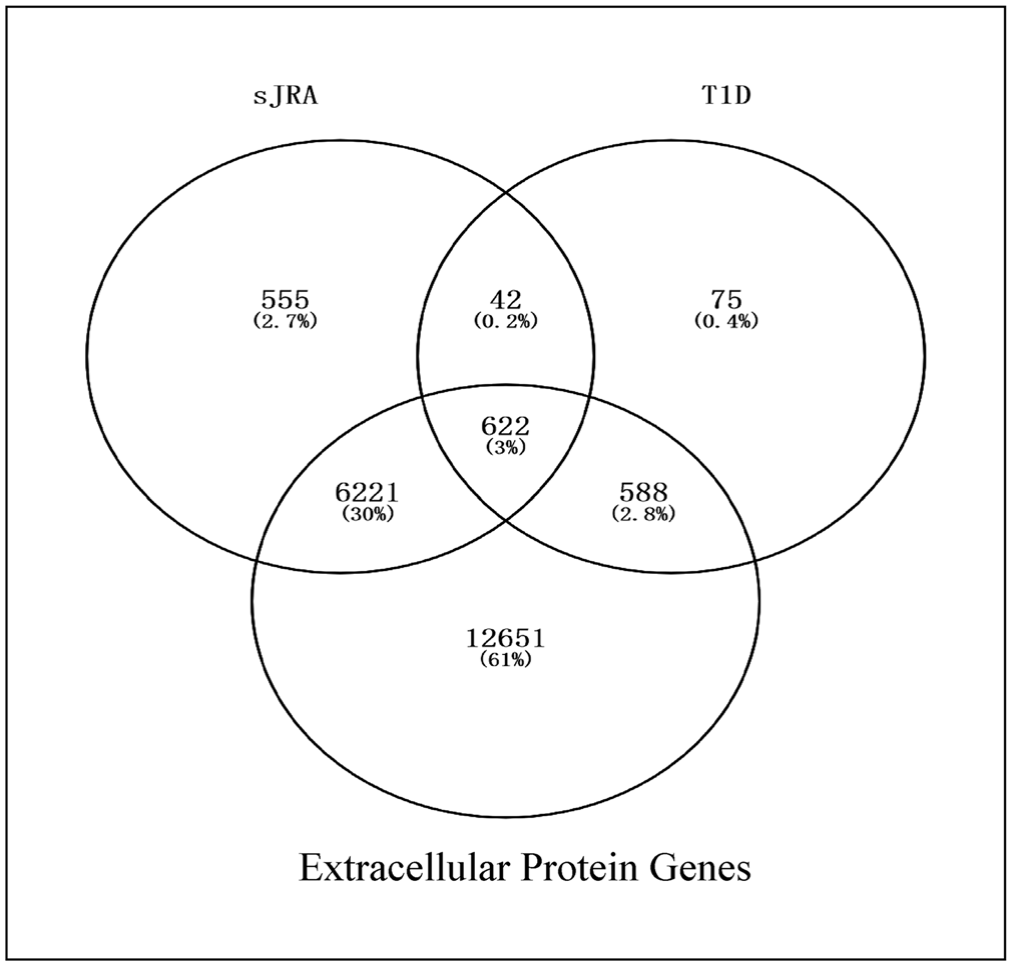
Relationship of genes of extracellular proteins and differentially expressed genes. *The percentage refers to ratios of genes in the circle to all genes listed.

As shown in Fig. 1, there were 175 up-regulated SDEGs and 245 down-regulated SDEGs in sJRA and T1D. Most of the DEGs are related to extracellular proteins (Fig. 2), and the vast majority of the SDEGs, except 6 of the up-regulated sDEGs and 9 of the down-regulated sDEGs, were extracellular protein-related genes (Supplementary Table S3).

### The shared transcription factors-SDEGs network construction

**Figure 3:**
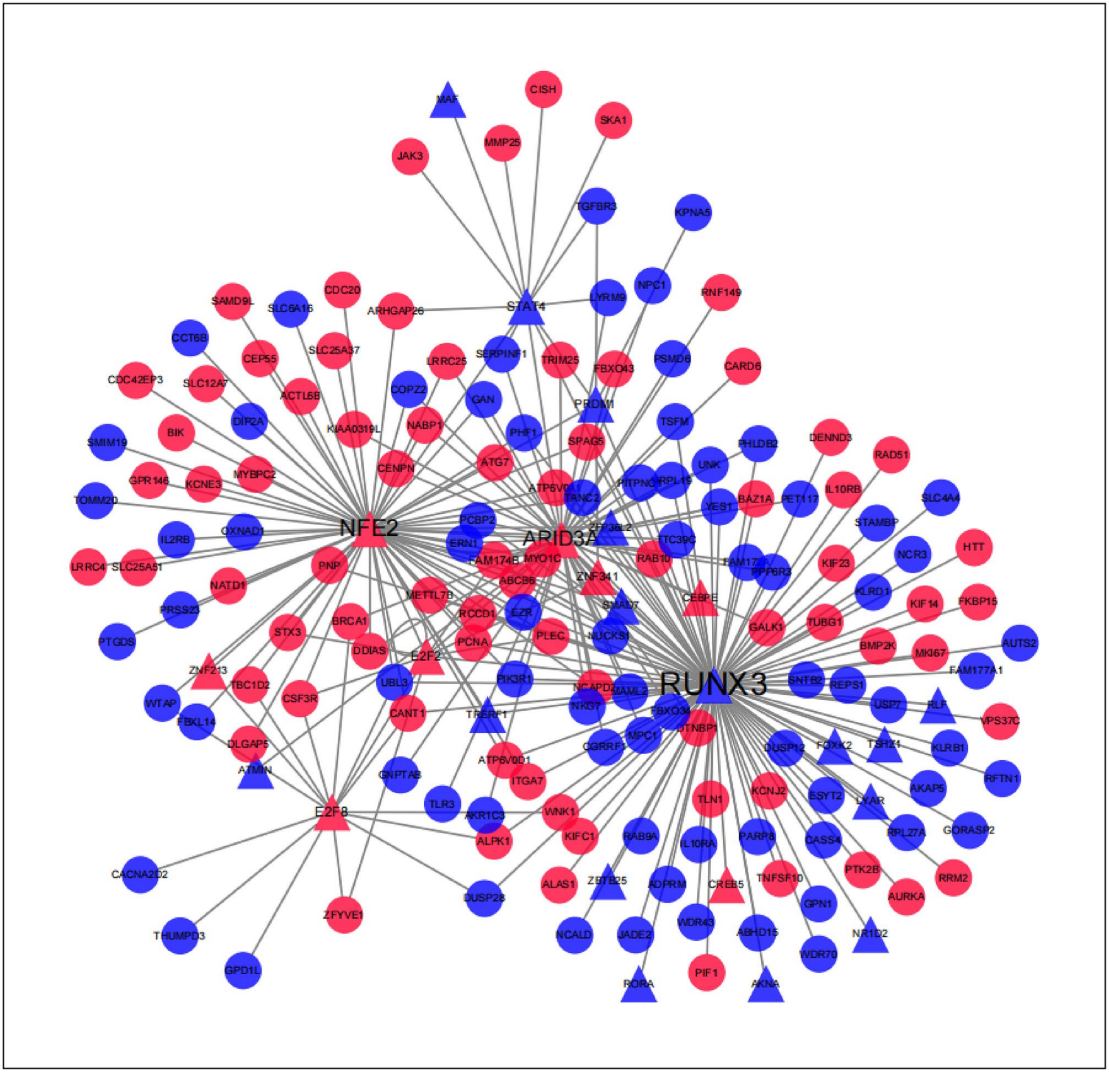
Shared transcription factors-SDEGs network. *Triangle* transcription factors, *Red* up-regulated SDEGs, *Blue* down-regulated shared SDEGs.

By comparing the data of HumanTFDB, 13 TFs in the up-regulated SDEGs (ARID3A, LTF, HLX, NFE2, E2F2, ZNF341, ZNF213, CEBPE, E2F8, MTF1, KLF17, THAP3, CREB5), and 40 TFs in the down-regulated SDEGs (ZNF8, ZSCAN25, MAF, ZNF256, PRDM4, ZIK1, AKNA, LYAR, NFATC2, ZBTB44, ZNF621, ZNF571, STAT4, ZNF329, L3MBTL4, ZXDB, RORA, ATMIN, SMARCE1, SMAD7, TRERF1, MYBL1, FOXK2, ZNF600, ZNF26, TSHZ1, BNC2, HOPX, ZNF568, ZFP36L2, RLF, ZNF28, ZNF684, ZBTB16, ZBTB25, NR1D2, ZNF254, ZXDA, RUNX3, PRDM1) were screened. As shown in Fig. 3, among those TFs, RUNX3 has the maximum potential target genes in the SDEGs (TFs-target SDEGs), ARID3A and NFE2 have the second and third most number of TFs-target SDEGs. As shown in the figure, the expression of RUNX3 is downregulated, while ARID3A and NEF2 are upregulated. Among these TFs, ARID3A and NFE2 are mutually target genes, besides, ARID3A is also regulated by RUNX3.

### The functional analyses of interested gene sets

Functional enrichment analysis of the up- and down-regulated SDEGs is shown in Supplementary Table S4. Up-regulated SDEGs were enriched in 238 GO terms, 62 REAC pathways, and 17 KEGG pathways. They are mainly involved in the cell cycle process, regulation of innate immune response, neutrophil degranulation, as well as several signaling pathways such as JAK-STAT, and PI3K signaling pathways.

The down-regulated SDEGs were only enriched in 154 GO terms, 36 REAC pathways, and 17 KEGG pathways. They are mostly related with adaptive immune system such as T cell activation, T/B cell receptor, Th1, Th2 and Th17 cell differentiation, and function of cytokines such as TGF-beta, interferon type I(IFN- I), interleukin(IL)23, IL12, IL11, IL2, IL3, IL5, etc. Besides, they were also enriched in innate immune system related terms such as natural killer cell function and signalings by M-CSF and GM-CSF.

**Figure 4:**
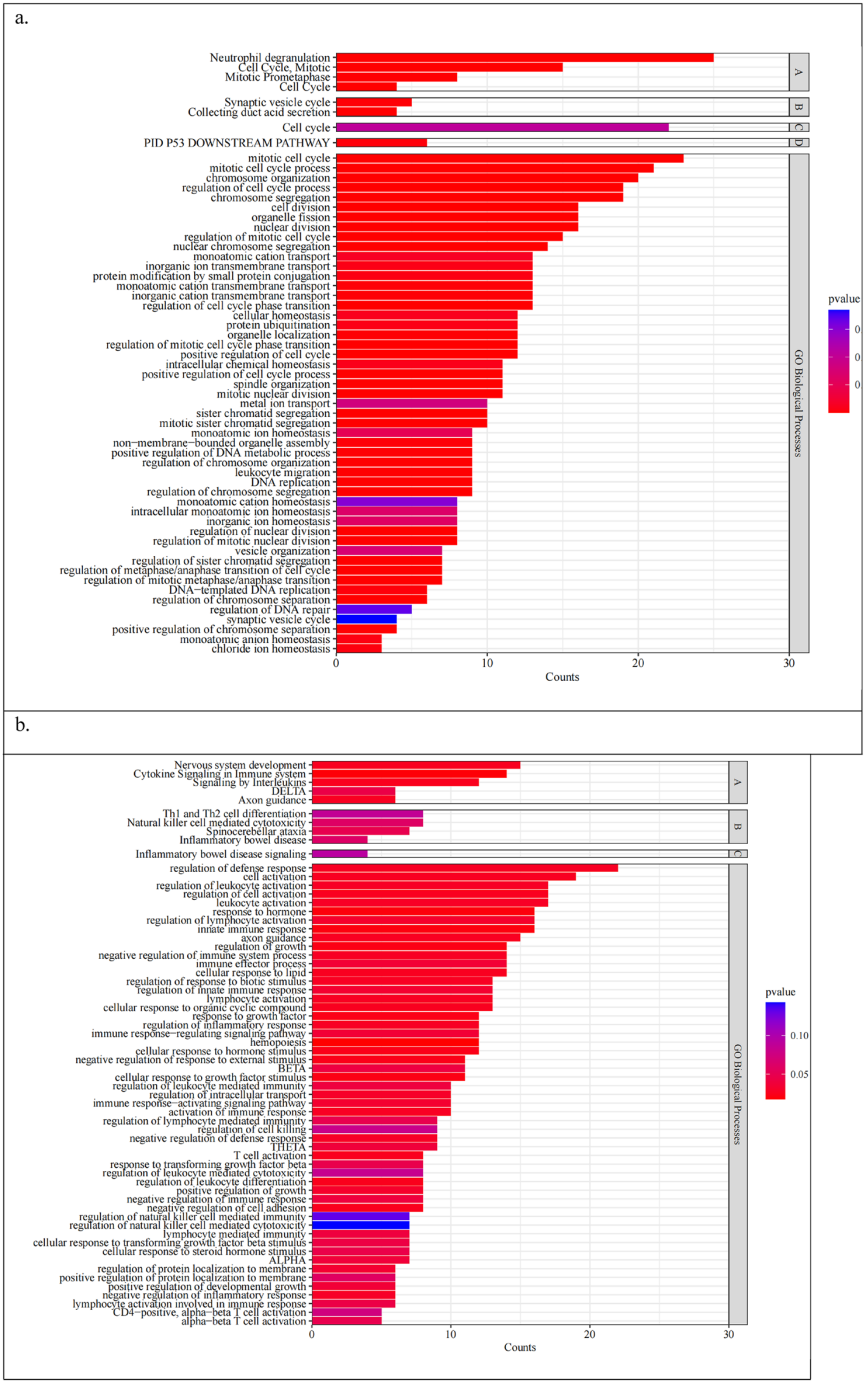
(**a**) The shared terms enriched between up-regulated SDEGs and TFs targeted SDEGs. (**b**) The shared terms enriched between down-regulated SDEGs and TFs targeted SDEGs. A: Reactome Gene Sets; B: KEGG Pathway; C: WikiPathways; D: Canonical Pathways; *ALPHA*: adaptive immune response based on somatic recombination of immune receptors built from immunoglobulin superfamily domains; *BETA:* immune response-regulating cell surface receptor signaling pathway; *THETA:* immune response-activating cell surface receptor signaling pathway; *DELTA:* immunoregulatory interactions between a Lymphoid and a non-Lymphoid cell.

The terms that up-regulated TFs targeted SDEGs enriched in are mostly included in the terms that up-regulated SDEGs were enriched in. The same in the enrichment results of down-regulated TFs targeted SDEGs. Figure 4a shows the shared terms enriched between up-regulated SDEGs and TFs targeted SDEGs. Still, neutrophil degranulation is closely related, together with cell cycle process. As is shown in Fig. 4b, the shared terms enriched between down-regulated SDEGs and TFs targeted SDEGs also involve CD4 positive T cell activation, Th1, and Th2 cell differentiation, cytokines functions as well as natural killer cell functions. Hormones feature like growth factor beta and steroid hormone stimulus were also involved.

### Immune cell infiltration in sJRA and T1D

**Figure 5:**
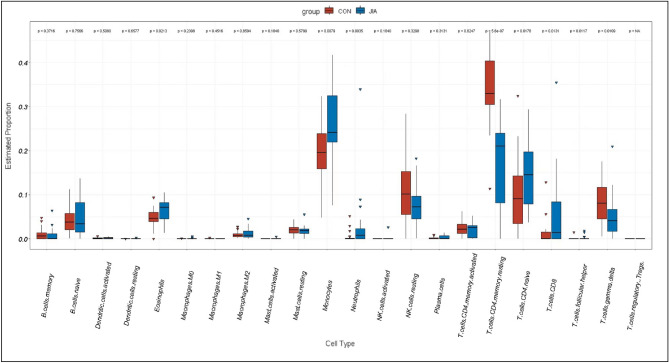
Comparison of 22 immune cells between sJRA samples and control samples.

**Figure 6:**
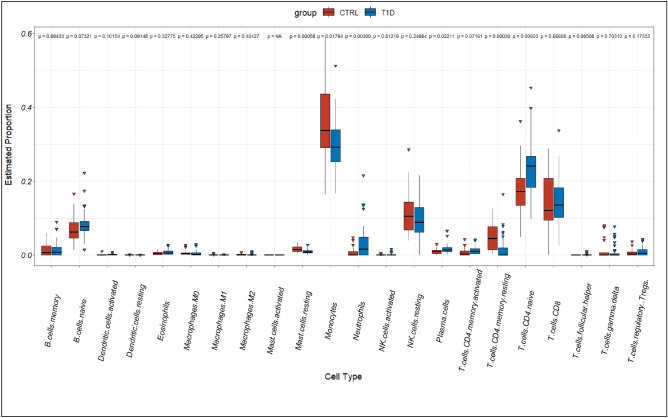
Comparison of 22 immune cells between T1D samples and control samples.

We performed CIBERSORT analysis to assess infiltrating levels of 22 immune cells in sJRA and T1D samples by R software. Our results showed that both diseases have higher levels of neutrophils, and CD4 naive T cells, while a lower level of CD4 memory resting T cells. Infiltrating levels of monocytes were also significantly different in both diseases compared to control samples. However, sJRA showed a higher level of monocytes (p < 0.05, Figs. 5, 6).

## Discussion

In our study, most of the SDEGs are extracellular protein-related genes. Extracellular proteins can have a wider range of effects in different biological processes. This also fits with the hypothesis that disorders of the immune system, in which cellular secretions play a key role, are important in sJRA and T1D. Up-regulated SDEGs were widely involved in biological processes such as cell cycle, regulation of innate immune response, and neutrophil degranulation, as well as JAK-STAT and PI3K signaling pathways. While down-regulated SDEGs were enriched in T cell activation, Th1, Th2 and Th17 cell differentiation, and functions of cytokines like TGF-beta. Infiltrating levels of immune cells also displayed the important role of CD4 positive T cell disorder.

Previous studies have found that major regulators of both acquired and innate immunity were upregulated in active sJRA patients, with upregulation of IFN-related genes and elevated serum IFN-γ levels^10–12^. The current view also suggests that T1D is a T cell-mediated autoimmune disease. The transcription of immune-related genes was more active and heterogeneous in T1D^13^. Its pathological process involves interactions between NK cells, different dendritic cell populations, and T cells that result in the attacking of pancreatic B cells and insufficient insulin levels^14^. The present results are consistent with previous reports and highlight the importance of these cells by bioinformatic analysis.

The TFs were identified in the SDEGs, and those who have the maximum potential target genes in the SDEGs were considered as key TFs. The functional analyses indicated that most TFs targeted SDEGs are enriched in the same terms that SDEGs are, suggesting the role of key TFs (ARID3A, NEF2 and RUNX3) and their regulatory networks in the pathogenesis jointly associated with sJRA and T1D. It also implies that the key TFs might be potential targets in the treatments.

The ARID family are DNA binding proteins, that have roles in embryonic patterning, cell lineage gene regulation, cell cycle control, etc. They also participate in the regulation of B-cell differentiation. ARID3A is closely related to IFN-α, and it has been found involved in the disease course of SLE by affecting the production of B cell-related autoantibodies^15, 16^. The high response of IFN-I and B cell disorders also exists in T1D and sJRA, suggesting a potential role of ARID3A in the pathogenesis of sJRA and T1D^17^. NEF2 can promote gluconeogenesis and induce hyperglycemia, and is also critical for regulating the maturation and differentiation of erythrocytes and megakaryocytes, and can affect the hyperplastic process of bone^18–20^. Different from ARID3A and NEF2, RUNX3 was downregulated in sJRA and T1D. It has been demonstrated that RUNX3 are important regulator of CD8 and CD4 T cell differentiation^21^ (35,490,198). RUNX3 has been found associated with the risk of JRA^22^. Although its mechanism has not been fully investigated, it might be related to the regulation of T cells. Previous study demonstrated that its participation in the pathogenesis of T1D are closely related with T cell receptor signaling and JAK-STAT signaling^23, 24^, and further influence IFNγ expression and Th1 phenotype^25^.

JAK-STAT is an evolutionarily conserved signaling pathway mediating cellular responses to cytokines and growth factors, mediating cell proliferation, differentiation, migration, apoptosis, and cell survival. The coordination of the MAPK-ERK and JAK-STAT pathways impacts growth hormone function and th1/th2 balance, which is also important in the pathogenesis of sJRA and T1D. Moreover, the JAK-STAT signaling pathway is involved in the pathological process of the sJRA-associated macrophage activation syndrome by influencing IFN, cytokine levels, and T cell activity^26–28^. Given the significant role of JAK-STAT in the regulation of metabolism and inflammation, its participation in diabetes is also being increasingly valued^29^.

Furthermore, we also found that innate immunity plays an important role in the shared pathogenesis of sJRA and T1D. The current view suggests that rheumatic and musculoskeletal disorders (RMDs) contain a spectrum of disorders, ranging from auto-inflammatory diseases dominated by innate immune disorders to autoimmune diseases dominated by adaptive immune disorders^30^. Many RMDs from within can be characterized by both innate and adaptive immune abnormalities. sJRA is one of the mixed types of RMDs that are biased toward innate immune abnormalities. Likewise, innate immune disorders also are believed to play an important role in T1D^31^. Neutrophils have a key role in innate immunity, and our results highlight that neutrophil functions like neutrophil degranulation are involved in the pathogenesis of sJRA and T1D^32^. Therefore, the roles of neutrophils warrant further investigation.

However, there remain insufficiencies in this research. Firstly, the information on the age (years) of the sJRA patients can be found in GSE7753 (15, 4.5, 14, 4.5, 4, 13, 11, 2, 3, 3, 14, 6, 1.5, 2, 5, 3.5, 3), but the age information in the control group is missing. The age range of the T1D patients in GSE193273 was 9.1–23.5 years, and that of the control group was 10.3–17.6 years. The age information was not clearly provided in other data sets. There may be bias in age-related gene expression. Secondly, the specific information on other related confounders, such as disease activity, drug use, and batch, was also incomplete. The underlying bias was not able to be controlled. Thirdly, when identifying targeted SDEGs of TFs, only those who have all epigenomic, ChIP-seq, and motif evidence were selected. It might lead to missing value because evidence of some targets was not fully verified.

In conclusion, our study suggests the complexity of the pathogenesis of sJRA and T1D, both of which jointly involve a wide range of biological processes. Our analysis supports the role of innate immune system like neutrophil degranulation disorder are also important in sJRA and T1D, as well as CD4 T cell functions and the JAK-STAT pathways, which are closely related to immune regulation. We also identified a set of candidate genes that work as TFs that may play regulatory roles in the complex network.

### Supplementary Information

Supplementary Tables.

## Data Availability

The datasets generated during and/or analysed during the current study are available in the GEO repository: https://www.ncbi.nlm.nih.gov/geo/query/acc.cgi?acc=GSE7753; https://www.ncbi.nlm.nih.gov/geo/query/acc.cgi?acc=GSE21521; https://www.ncbi.nlm.nih.gov/geo/query/acc.cgi?acc=GSE193273; https://www.ncbi.nlm.nih.gov/geo/query/acc.cgi?acc=GSE55100; https://www.ncbi.nlm.nih.gov/geo/query/acc.cgi?acc=GSE9006;
